# Performance Analysis of Satellite Missions for Multi-Temporal SAR Interferometry

**DOI:** 10.3390/s18051359

**Published:** 2018-04-27

**Authors:** Fabio Bovenga, Antonella Belmonte, Alberto Refice, Guido Pasquariello, Raffaele Nutricato, Davide O. Nitti, Maria T. Chiaradia

**Affiliations:** 1 Research National Council of Italy, ISSIA, 70126 Bari, Italy; belmonte@ba.issia.cnr.it (A.B.); refice@ba.issia.cnr.it (A.R.); pasquariello@ba.issia.cnr.it (G.P.); 2 GAP S. R. L., 70125 Bari, Italy; raffaele.nutricato@gapsrl.eu (R.N.); davide.nitti@gapsrl.eu (D.O.N.); 3 Dipartimento di Fisica “M. Merlin”, Politecnico di Bari, 70125 Bari, Italy; chiaradia@ba.infn.it

**Keywords:** SAR, Multi-temporal/Multi-sensor SAR Interferometry, Ground displacement monitoring

## Abstract

Multi-temporal InSAR (MTI) applications pose challenges related to the availability of coherent scattering from the ground surface, the complexity of the ground deformations, atmospheric artifacts, and visibility problems related to ground elevation. Nowadays, several satellite missions are available providing interferometric SAR data at different wavelengths, spatial resolutions, and revisit time. A new and interesting opportunity is provided by Sentinel-1, which has a spatial resolution comparable to that of previous ESA C-band sensors, and revisit times improved by up to 6 days. According to these different SAR space-borne missions, the present work discusses current and future opportunities of MTI applications in terms of ground instability monitoring. Issues related to coherent target detection, mean velocity precision, and product geo-location are addressed through a simple theoretical model assuming backscattering mechanisms related to point scatterers. The paper also presents an example of a multi-sensor ground instability investigation over Lesina Marina, a village in Southern Italy lying over a gypsum diapir, where a hydration process, involving the underlying anhydride, causes a smooth uplift and the formation of scattered sinkholes. More than 20 years of MTI SAR data have been processed, coming from both legacy ERS and ENVISAT missions, and latest-generation RADARSAT-2, COSMO-SkyMed, and Sentinel-1A sensors. Results confirm the presence of a rather steady uplift process, with limited to null variations throughout the whole monitored time-period.

## 1. Introduction

Since 2000, several Multi-temporal InSAR (MTI) algorithms for the detection of ground surface deformation signals have been developed and applied to study different geophysical phenomena [1,2,3,4]. MTI algorithms can be grouped into two categories: Persistent Scatterers (PS) Interferometry (PSI) [5], relying on the phase information from single isolated objects characterized by a high temporal phase stability, and Small Baseline Subset (SBAS) [6] exploiting distributed scatterers (without any dominant element within the resolution cell), which are more sensitive to both temporal and volume decorrelation than PS. Recently hybrid approaches (e.g., [7,8]) have been proposed, to include both single and distributed scatterers. Differences in number and spatial distribution of detected targets can occur, depending on the scattering characteristics of the ground surface. MTI applications pose challenges related to:

- unfavorable settings of the area due to, for example, vegetation or variable land cover (relevant for applications relating to subsidence, landslides and tectonics), and/or steep topography (relevant for landslide applications);
- kinematics of the phenomenon, e.g., in case of strong nonlinearity (relevant for subsidence or landslides), or extremely slow deformation trends (relevant for tectonics);
- size of the phenomenon, that can be very local, e.g., for small landslides, or very large, e.g., for tectonics;
- atmospheric and orbital artifacts, which depend on the wavelength and orbital precision, and affect all possible applications, but in particular, tectonic studies at regional scale.

Nowadays, several satellite missions are available, providing interferometric SAR data at different wavelengths, spatial resolutions, and revisit times. In Table 1, the principal satellite missions are listed with those parameters which are relevant for the performance analysis dedicated to ground deformation applications. High-resolution X-Band SAR sensors, such as the COSMO-SkyMed (CSK) and TerraSAR-X (TSX) constellations, acquire data with spatial resolution reaching metric values, and provide revisit times of up to a few days, leading to an increase in the density of the measurable targets, as well as to improvements in the detection of nonlinear displacements. Medium resolution C-band SAR data have been thoroughly exploited in the last two decades, thanks to the ERS-1/2 and ENVISAT-ASAR (ENV) missions, and RADARSAT-1/2 (RSAT1/2). A recent interesting opportunity is provided by the Sentinel-1 (S1) constellation [9], which has a spatial resolution comparable to previous ESA C-band missions, and a revisit time reduced to 6 days, by using the two satellites. By offering regular global-scale coverage, improved temporal resolution, and freely available imagery, S1 guarantees an increasing use of MTI for ground displacement investigations.

According to these different SAR space-borne missions, the present work discusses current and future opportunities of MTI applications to ground instability monitoring. Issues related to coherent target detection, mean velocity precision, and product geo-location are addressed through a simple theoretical model, assuming backscattering mechanisms related to a strong coherent scatterer. In particular, a comparative analysis is carried out, aimed at addressing specific advantages and limitations of different satellite missions.

The paper also presents an example of multi-sensor ground instability investigation over the Lesina Marina site in Southern Italy, a tourist village lying over a gypsum diapir, where a hydration process, involving the underlying anhydride, causes a smooth uplift pattern affecting the entire village area, and the formation of scattered sinkholes, which add a threat to the village buildings integrity. More than 20 years of MTI SAR data have been used, coming from both legacy ERS and ENV missions, as well as state-of-the-art RSAT2, CSK, and S1 sensors. Displacement maps from the different sensors are compared in terms of the density of measured coherent targets and the precision of the estimated mean velocity.

## 2. Performance Analysis

The mission parameters, which impact on the quality of the MTI products, are the wavelength of the SAR signal (decreasing from L, ~23 cm, to C, ~5.5 cm, to X band, ~3.1 cm), the revisit time, the spatial resolution, and the orbital tube. The figures that allow estimating the quality of the displacement maps are: the number of measurable coherent targets (CT) on the grounds (e.g., the PS density), the maximum detectable displacement velocity, the minimum number of processed images (*N*) or the observation time span (*T*) needed to derive reliable products, and the noise affecting both the final displacement and residual height estimation. 

The density of detectable targets depends heavily on the ground cover, being in general highest over urban areas, thanks to the abundance of artificial structures with coherent backscattering. Both wavelength and spatial resolution can impact on the CT density. Several studies have proven that high-resolution data from CSK, TSX, and RSAT2 lead to a significant increase in the number of potential CT with respect to the data from medium resolution missions, such as ERS1/2, ENV, and RSAT1. Regarding the X-band sensors, CT density increases from about five to more than ten times, depending on the land use, with very high densities (up to ~70,000 CT/km^2^) in case of bare soil [3]. Similar results are reported by using C-band RSAT2 data acquired in “Fine” mode (3 m resolution). There is limited information in the literature on the PS densities obtainable from L-band MTI, due to the few datasets available over Europe and suitable for MTI. Finally, short revisit times and long wavelengths (S1) allow detection of signals from targets with short de-correlation time, such as distributed scatterers. For example, the study presented in [10] shows MTI over a pasture, estimating for C-band a de-correlation time of about 10 days or less (depending on the data resolution); in these conditions, while the use of ENV data is unfeasible, S1 data (revisit time of 6 days) should be able to improve the number of measurable targets on the ground.

Another useful figure for assessing the limits of MTI application ground displacement monitoring is the maximum measurable velocity. Since interferometric phase samples are known only with modulo 2π (wrapped phase), it is not possible to measure phase differences >π unambiguously, which correspond to line-of-sight (LOS) deformation >λ/4. This constrains the measurable displacement velocity. Since MTI procedures compute phase differences both in space (between PS pairs) and time (between consecutive acquisitions), the maximum detectable velocity depends on: (i) the spatial gradient of the deformation; (ii) the CT spatial sampling, which depends on the CT spatial density; (iii) the radar wavelength; (iv) the temporal sampling of the acquisitions defined by the revisit time. Note that the first two factors depend on the particular phenomenon and environmental setting being investigated, and thus, are not known a priori. Table 1 reports, for each SAR mission, the maximum detectable velocity between neighbouring CT, which can be considered as a lower bound. With shorter wavelengths, the measurable velocities decrease, but at the same time the sensitivity to LOS displacements is increased, leading to the possibility of detecting very low (mm/year) displacement rates with high precision. This is of practical significance for, for example, the timely detection of pre- and post-failure movements affecting land or infrastructures. Moreover, the velocity constraint, which is also influenced by the revisit time, can be considerably relaxed using data from the latest X-band missions, in particular the CSK constellation, which reduced the revisit time to a few days, thus counterbalancing the negative effect of the short wavelength. This implies the possibility of detecting higher maximum velocity values by using X-band data, rather than C/L-band data, at least for the currently available SAR missions (see Table 1). The performance of C-band MTI is improved thanks to the recently lunched S1 mission, that responds to the specific requirements of InSAR monitoring, providing revisit times of 6 days.

### 2.1. Mean Velocity Precision

Concerning the noise affecting the precision of displacement measurements, a simple theoretical model was developed which provides the velocity standard deviation ($\sigma_{v}$) as a function of the number of acquisitions (N+1), the wavelength (λ), the spatial resolution along ground range and azimuth directions ($\Delta X_{gr}$, $\Delta X_{az}$), the revisit time (dt), the clutter-normalized radar cross section ($\sigma_{0}$), and the average residual atmospheric phase noise. The model assumes a PSI processing scheme of a single-master interferometric stack (N interferograms), derived from SAR acquisitions which are regularly sampled in time. In these hypotheses, the displacement estimation can be modeled as a linear regression in the presence of a normally-distributed, white process, and the mean velocity standard deviation computed as [11,12]:

(1) $$\sigma_{v}=\sqrt{\frac{12}{N\left(N^{2}-1\right)dt^{2}}}\cdot \frac{\lambda }{4\pi }\sigma_{\varphi }$$

The phase noise $\sigma_{\varphi }$ affecting the final estimation depends on both the backscattering ($\sigma_{SCR}$) of the PS resolution cell (related to the pixel interferometric multi-temporal coherence), and the residual atmospheric phase signal ($\sigma_{atm}$) resulting from the filtering performed by the multi-temporal analysis. The latter can be assumed to depend on the ratio between the two-way travel path $\sigma_{dr,atm}$ (independent from the wavelength and approximated to 1 cm) [13,14] and N. In order to model the noise level related to the backscattering of the coherent target, we refer to the case of a dominant scatterer in a weak clutter, showing high Signal to Clutter Ratio (SCR). The phase noise related to the backscattering properties, $\sigma_{SCR}$, can be expressed as a function of the interferometric coherence [15], which, in the hypothesis of a very coherent target, is related to the SCR [16], that we compute in this case for a triangular trihedral corner reflector (of edge length $l_{CR}$) in bare soil clutter, with a normalized radar cross section $\sigma_{0}$. According to this, we can express the phase noise as:

(2) $$\sigma_{\varphi }^{2}=\sigma_{SCR}^{2}+\sigma_{atm}^{2}=\frac{1}{2\cdot SCR}+{\left(\frac{2\pi }{\lambda }\sigma_{dr,atm}\right)}^{2}=\frac{1}{2}\left(\Delta X_{gr}\cdot \Delta X_{az}\cdot \sigma_{0}\right)\frac{3\lambda^{2}}{4\pi \cdot l_{CR}^{4}}+{\left(\frac{2\pi }{\lambda }\right)}^{2}\frac{\sigma_{dr,atm}^{2}}{N}$$

**Figure 1:**
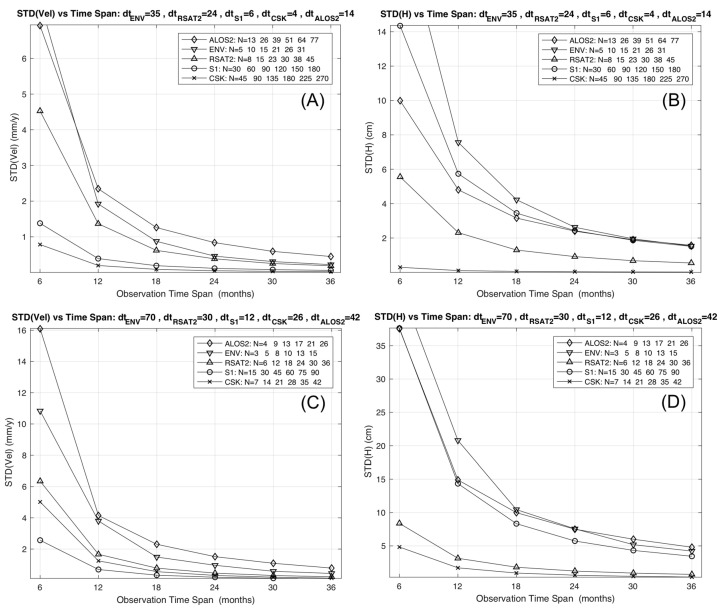
Mean velocity standard deviation (**A**,**C**) and lower limit of the height standard deviation (**B**,**D**) corresponding to different observation time spans, and computed for ALOS2, ENV, RSAT2, CSK and S1 missions, according to revisit time values reported in the respective plot titles.

By combining (1) and (2), the velocity standard deviation can be expressed as a function of geometrical and radiometric parameters, thus allowing the evaluation of the performance of the MTI for different satellite missions. Once the target size and the characteristics of the soil (on which the clutter noise depends) are defined, the model simulates a pixel with a coherence value, which depends on the spatial resolution and wavelength of the different SAR missions. The phase noise decreases as the wavelength decreases and the resolution increases, while the error on the final velocity estimation depends also on both the number of acquisitions and the investigated time span (*T*). Plots A and C in Figure 1 show the velocity standard deviation derived by the model for different observation time spans and for different satellite missions: ALOS-2 L-band mission, ENV, RSAT2 and S1 C-band missions, and CSK X-band mission. The incident angle has been set to 35° and the $\sigma_{0}$ was calculated for L, C and X bands, assuming a bare soil roughness with a height standard deviation of 0.9 cm, and a correlation length of 10 cm. Two configurations of revisit times (dt) have been tested and reported in the plot title: one (A) with the minimum nominal values, the other (C) with higher values, corresponding for ALOS2 to 42 days, and for ENV, RSAT2, S1 and for CSK to the mean revisit times of the real datasets used in the following (see Table 2). The number of acquisitions changes according to the values set for the revisit time and the observation time span, as reported in the legend of each plot. Performance bounds of other missions either present (e.g., Gaofen-3, KOMPSAT-5) or future (e.g., Tandem-L, RADARSAT Constellation Mission, COSMO-SkyMED-SG, TerraSAR-X-NG) can be deduced from the plots, by accounting for the changes in the system parameters.

The model shows that the velocity standard deviation decreases as the observation time span becomes longer, since the number of acquisitions involved in the estimation process increases. This trend is modulated by the SAR mission parameters, so that the absolute value of the mean velocity precision becomes suitable (i.e., below a reliable threshold) for different values of *T* or *N*, depending on the SAR mission. In particular, as higher resolutions and shorter revisit times are considered, reliable estimates of the displacement rates are possible (i) by using fewer SAR scenes for the same time span, or (ii) by using the same number of images but acquired in a shorter time span. As can be seen, the CSK and S1 constellations clearly outperform the others in both terms, and S1 offers significantly improved performances with respect to previous C-band missions.

### 2.2. Height Precision

A similar model can be used to derive the standard deviation of the residual height estimation, which is a standard product of the MTI algorithms, impacting on the final geo-location precision of the detected targets. The residual height is estimated through a linear regression along the interferometric normal baselines, $b_{⊥}(n)$. Assuming a Gaussian distribution for the normal baselines, the height standard deviation can be computed as:

(3) $$\sigma_{h}=\sqrt{\frac{1}{{\left(N\sigma_{b_{⊥}}\right)}^{2}-N{\langle b_{⊥}\rangle }^{2}}}\cdot \frac{\lambda R_{0}sin(\theta )}{4\pi }\sigma_{\varphi },$$

where $R_{0}$ is the nominal value for the near range, and $\langle b_{⊥}\rangle$ and $\sigma_{b_{⊥}}$ are the mean and standard deviation of the baseline distribution respectively. Several tests were carried out, by exploring SAR datasets from ERS, ENV, CSK and S1, in order to derive values representative of the normal baseline distributions. While a zero-mean distribution can be generally considered a reliable approximation for all missions, the baseline standard deviation depends on the dataset at hand, so that it is difficult to derive a single value characterizing each mission. In order to overcome this problem, the maximum baseline value can be considered, in lieu of the baseline dispersion. It can be computed as the mission orbital tube diameter ($D_{orb}$), and allows deriving a lower limit for the height standard deviation:

(4) $$\sigma_{h}=\frac{\lambda R_{0}sin(\theta )}{4\pi }\frac{\sigma_{\varphi }}{N\cdot \sigma_{b_{⊥}}}\geq \frac{\lambda R_{0}sin(\theta )}{4\pi }\cdot \frac{\sigma_{\varphi }}{N\cdot D_{orb}}$$

By combining (2) and (4), the height standard deviation lower limit can be derived as a function of geometrical and radiometric parameters. Plots B and D in Figure 1 sketch the lower limit computed for ALOS2, ENV, RSAT2, S1, and CSK, and for different observation time spans. We used the nominal value of the orbital tube diameter derived from the literature [17,18,19,20,21] and listed in Table 3. Two configurations of revisit times (dt) have been tested and reported in the plot title: one (B) with the minimum nominal values, the other (D) with higher values, corresponding for ALOS2 to 42 days, and for ENV, RSAT2, S1 and CSK to the mean revisit times of the real datasets used in the following (see Table 3).

Again, the height standard deviation decreases as the observation time span becomes longer, since the number of acquisitions involved in the estimation process increases, and this trend is modulated by the SAR mission parameters. For any timespan, the expected height standard deviation is lowest for CSK. In contrast, the S1 performance in terms of height estimation is decreased with respect to the one related to the mean velocity, due to its narrow orbital tube; this impacts negatively on the geo-location capability of the mission.

The proposed model provides a closed formulation without the need of simulations or more complex hypotheses on the characteristic of the interferometric coherence. This choice confines the applicability of the model to the PS scatterers and related approaches, and it is consistent with the results presented in the following section for the test case derived through a PSI processing. In order to also consider distributed scatterers, the impact of temporal de-correlation has to be included in the model. MTI algorithms dealing with this kind of targets basically explore all the interferograms derivable from the available images in order to select those with reliable coherence values. Different strategies can be adopted (e.g., [6,7,11]) to select and estimate the parameters of interest. Regardless, the final estimation depends on the number of interferograms M≤N(N+1)/2 (for N+1 images), and on de-correlation phase noise affecting each interferogram. According to [11], this contribution is dominant when dealing with a large number of images, since in this case it is possible to achieve a robust estimation and removal of the atmospheric noise, which thus becomes negligible. Although a final closed form can be derived (as in [11]), this still depends on parameters modeling the temporal decorrelation, which are hardly generalizable. The proposed model, although confined to a PS targets scenario, provides a simple formulation, which depends on radiometric and geometric parameters that can be easily retrieved for any satellite mission.

Furthermore, we assume ideal PSI processing with reliable removal or atmospheric and orbital artifacts, and no aliasing of the interferometric phase both in space and time. In case of real, harsh and complex scenarios, the values provided by the model could slightly overestimate the actual precision (for both mean velocity and residual height). However, this does not affect the comparative performance analysis between different configurations or missions. A general conclusion is that for all the simulated missions and configurations, observation times longer than 18 months should be able to guarantee reliable precisions for both mean displacement rates (less than 2 mm/year velocity error) and geo-location (roughly less than 10 cm vertical error). Moreover, by considering the forthcoming new SAR missions with interferometric capabilities (e.g., SAOCOM, Tandem-L, NISAR, RADARSAT Constellation Mission, PAZ, COSMO-SkyMED-SG, TerraSAR-X-NG), the resolutions and revisit times will be able to further improve these performances.

## 3. Results over a Sample Test Site

**Figure 2:**
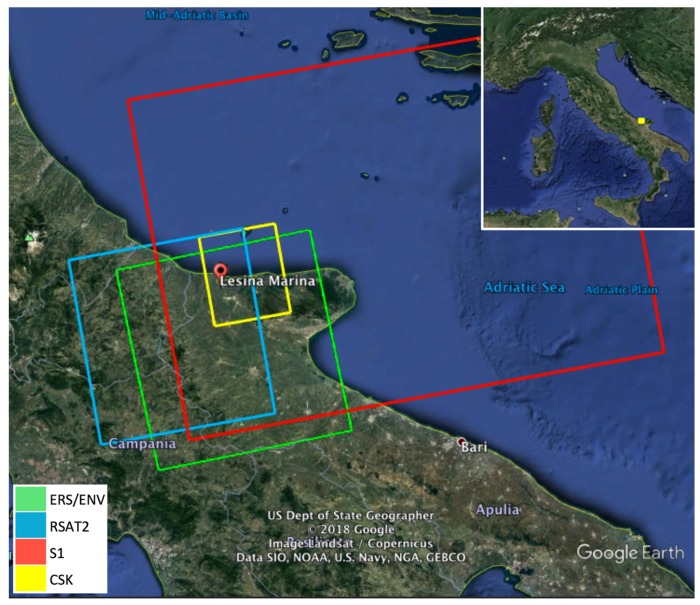
Ground coverage of the SAR datasets used for the MTI processing over Lesina Marina.

In order to provide an example of a multi-sensor ground instability investigation, we present the test case of Lesina Marina, in Southern Italy, a site affected by interesting ground displacement phenomena, where several SAR datasets are available, acquired along ascending orbits from ERS, ENV, RSAT2, CSK and S1 satellite missions, and covering more than 20 years with varying ground resolutions, frequency bands and repeat times. Figure 2 and Table 2 provide, for each dataset, details about the SAR frame ground coverage, the number of acquisitions, and the observation time span.

**Figure 3:**
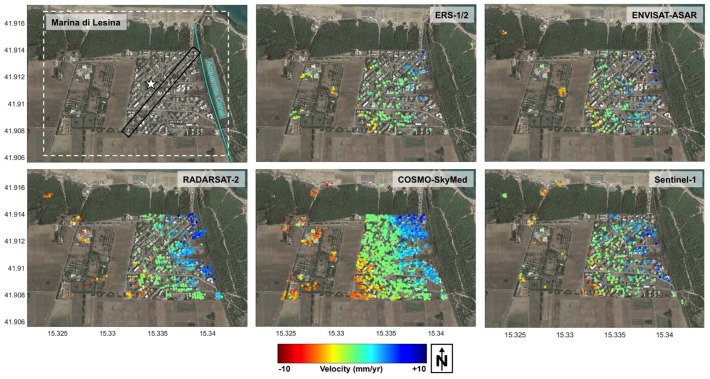
PS average LOS velocity maps for the Lesina Marina area: ERS, ENV, RSAT2, S1 and CSK. All the time series are acquired in ascending mode, right-looking configuration, with slightly different incidence angles (listed in Table 2). The white star in upper-left plot indicates the location of the reference PS point, common to all datasets. The black oblique rectangle is used to extract the PS velocity profiles shown in Figure 4.

The Lesina Marina village (see Figure 2) sits on a diapir made of Triassic gypsum, mantled by Quaternary sandy deposits. It is a peculiar geological site, affected by uplifting and sinkhole phenomena, causing instabilities and failures of infrastructures. The cutting, in 1930, of the artificial Acquarotta canal (sketched in light blue in the top-left map in Figure 3), connecting the nearby Lesina lagoon to the Mediterranean Sea, exposed this grey micro and meso-crystalline gypsum. This event is a likely cause for the formation of the dissolutional conduits and cavities found in the area, leading to the formation of sinkholes, which have been plaguing the site in recent years [22].

The MTI monitoring covers a time period starting from 1995, with legacy ERS and ENVISAT sensors, and extending to 2016, with data including RSAT2, CSK and S1. Early results showed the unexpected presence of steadily uplifting PS objects over the Lesina Marina urban area, with maximum values located around the Acquarotta canal, exhibiting average uplift velocities reaching 4–5 mm/year, and decreasing smoothly towards the W-SW to about zero, with locally slightly negative values. These uplift rates, validated by using leveling measurements [23], overcome the value of about 1 mm/year which was estimated on the diapir detected underneath the Lesina Marina area. This has been attributed to a hydration phenomenon likely caused by the exposition of the gypsum core, with residual anhydrite inside, to the water coming from the canal [23]. Uplift velocities and geometry are compatible with the presence of anhydrite hydration, which has been shown to result in increases in volume of up to 60% [24].

The mean LOS deformation velocity maps derived from the MTI processing of the various sensors time series are reported in Figure 3. We used two independent PSI processing chains, SPINUA [25,26] and StaMPS [8,27], with fully comparable results. PS populations are selected by applying suitable thresholds to the temporal coherence [28], with a merit figure quantifying the residual noise in each point displacement estimate. High-resolution data actually increase the density of measurable targets on the ground, thus improving the delineation of the spatial deformation pattern, and increasing the chances of capturing signals related to local instabilities. The average PS density computed within the area enclosed in the white dotted rectangle in Figure 3 increases from about 100 PS/km^2^ for ERS, ENV and S1 data, to about 200 PS/km^2^ for RSAT2, and finally to more than 1000 PS/km^2^ in the case of CSK data.

**Figure 4:**
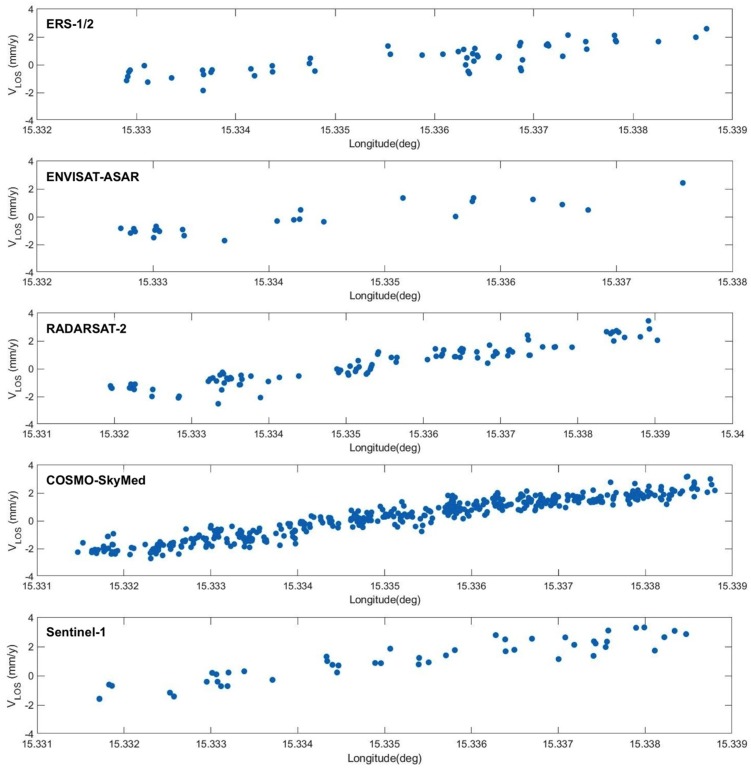
Profiles of the PS velocity computed along the rectangle sketched in black in the upper left plot of Figure 3. The plots correspond, from top to bottom, to ERS, ENV, RSAT2, CSK, and Sentinel-1.

PS data from all the time series were precisely geo-referenced. Neglecting very local movements, close PS points can be assumed to share a common movement history. Results from all datasets provide similar deformation patterns, showing an uplift that decreases smoothly towards W-SW, thus confirming previous results from ERS and ENVISAT datasets presented in [23]. This common spatial trend of deformation is captured by the PS velocity profiles in Figure 4, computed within the rectangle sketched in black in the upper left plot of Figure 3. The plots correspond, from top to bottom, to ERS, ENV, RSAT2, CSK, and Sentinel-1 datasets.

**Figure 5:**
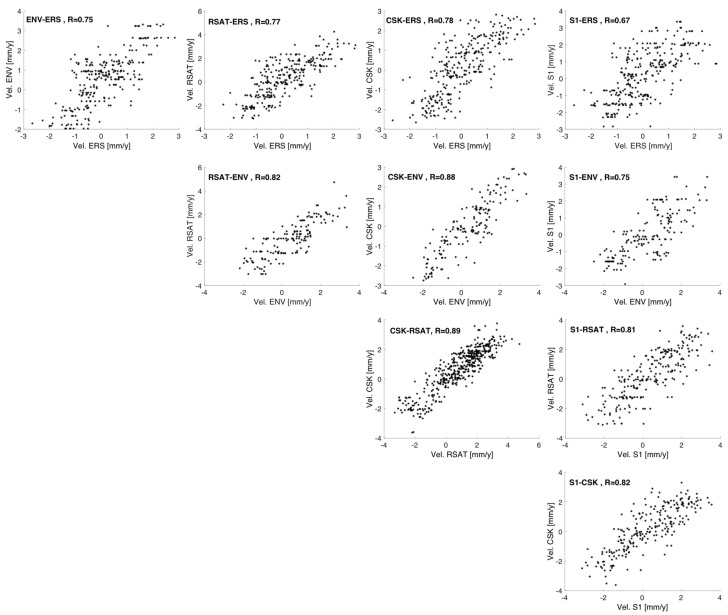
Scatterplot of the difference between vertical components of the average LOS velocities derived from the datasets in Figure 3.

In order to compare the PS populations having different ground resolutions, locations, and numbers of PS targets, we then assumed the ground displacement pattern to be constant throughout the entire monitored time interval, and selected, for each pair of datasets, the common PS targets as those lying within a distance of 100 m. Figure 5 sketches, for each pair of datasets, the scatter plot computed by using the mean velocity values derived from the common PS targets, and projected along the vertical direction. We derived horizontal and vertical components in previous work [23] by combining ascending and descending datasets from ERS and ENVISAT datasets. Results showed that the movement is mostly vertical (in particular close to the canal). According to this, in the present case, where only ascending acquisitions are available for all the sensors, we neglect the small horizontal component, which allowed us to approximate the computation of the mean velocity vertical component as the ratio between the LOS component and the cosine of the incident angle. This approximation allows a normalization of the results of the various datasets with respect to their different incident angles. Table 4 reports the mean and standard deviation of the difference between the vertical components of the mean velocity values for each pair the correlation coefficient. Velocity distributions do not show significant differences. The values are bound in the interval [−2, 4] mm/year, confirming that the uplift phenomenon has been going on rather steadily for the last 20 years. This is further confirmed by the correlation coefficients ranging from 0.67 and 0.89, as well as by the values of the mean and standard deviation of the differences, which are mostly within 1 mm/year.

**Figure 6:**
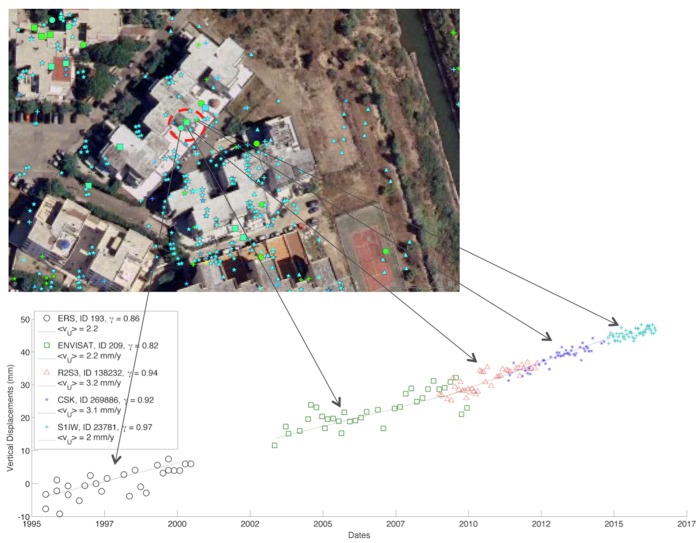
Temporal trend of the vertical displacement corresponding to the area on the ground delimited by the red dotted circle. Displacement values from different sensors are connected together through a minimum-norm paradigm.

In order to analyze not only the mean velocity values, but also the displacement values, in Figure 6 we show an example of displacement time series derived by combining the results of all the datasets corresponding to the same area on the ground. LOS displacements and mean velocities have been projected on the vertical direction before integration. The vertical displacement values from the different sensors were then connected together through a minimum-norm paradigm, i.e., assuming the ensemble time series can be approximated with a low-order polynomial or a piecewise linear function. More complex approaches [29] could help in case of the presence of strongly nonlinear motion on natural terrain exhibiting low temporal coherence conditions. The final displacement trend covers more than 20 years, and confirms the linear behavior of the uplift. As expected, the noise affecting the displacements changes according to the differences in wavelength, resolution, and number of acquisitions, decreasing from the former to the most recent missions. Moreover, thanks to the short revisit times, CSK and S1 are potentially suitable to also measure strong nonlinear trends and the related accelerations. These characteristics appear useful in the investigation of the complex phenomena of sinkholes, which affected recently Lesina Marina.

## 4. Discussion

According to the results derived by parametric analysis, in the following paragraphs we discuss potentials and limitations of the different SAR missions with respect to mission parameters and applicative domains.

The mission parameters considered for the performance analysis are: the wavelength, the spatial resolution, the revisit time, and the orbital tube size. Some considerations derive directly from the properties of the interaction between SAR signal and ground surface, while others require taking into account the concurrent effects that the mission parameters have on the quality of the MTI results. These can be related to the precision of both mean velocity and residual height, and were estimated by using a model developed ad hoc.

The wavelength is inversely proportional to the displacement sensitivity, and therefore the use of small wavelengths (X band) is advisable in applications requiring the measurement of very slow movements (e.g., monitoring of landslides or tectonic faults).

Small revisit times and longer wavelengths (S1) allow relaxing the constraint on the spatial and temporal distance between the measurements needed to avoid aliasing and to perform reliable phase unwrapping. This impacts on the monitoring of subsidence phenomena and landslides in particular. For example, the detection of changes in the kinematic regime (rapid and non-linear) is related to signals preceding the loss of the equilibrium conditions on unstable slopes. Moreover, a short revisit time in general guarantees good interferometric coherence, and allows the acquisition of SAR images close to the event. This is crucial, for mapping ground deformations related to the main shock of an earthquake, for instance.

The accuracy on the mean displacement rates depends on wavelength, spatial resolution (which impacts on the phase noise), number of acquisitions, and the observation time-span (which depends on the revisit time), while the accuracy of the height measurement, which determines the quality of the product geocoding, depends also on the orbital tube size. The proposed model shows how the X band, and in particular the CSK constellation, provides the best performance in terms of accuracy on the estimated mean velocity, especially for limited observation time spans (see plot A in Figure 1). This improves the monitoring capabilities in high-risk situations by providing results in a shorter time, and allows the monitoring of, for example, mountainous areas during short periods, avoiding snow coverage, thus maximizing the chance of detecting coherent targets. Moreover, short revisit times allow us to collect large data stacks in short times, and to improve temporal sampling, thus increasing the chances of catching high-rate, nonlinear signals. For longer observation spans, the differences with respect to C band missions become negligible. Therefore, missions with characteristics similar to the CSK constellation in X band are particularly advantageous for applications in emergency scenarios to capture signals preceding or following events (e.g., landslides, earthquakes, collapses) characterized by strong non-linearity. For phenomena characterized by very slow and steady kinematics that require analysis of long time intervals (tectonics, volcanoes), C-band missions are competitive and effective. S1 greatly improves the performance with respect to the other C-band missions, since the low spatial resolution is compensated by the short revisit time. However, the geocoding accuracy of this mission is compromised by the small size of the orbital tube, which reduces the accuracy of the estimated target height. This represents a limit for applications that require the identification and monitoring of single structures affected by displacement.

X-band sensors, by providing better spatial resolutions than those operating in other bands, improve the signal-to-noise ratio on resolution cells characterized by point-like backscattering, and thus considerably increase the density of the measurable PS targets on the ground. In contrast, small revisit times and long wavelengths allow us to extend the measurements to distributed scatterers, characterized by a homogeneous distribution within the resolution cell, whose interferometric response has a de-correlation time that depends directly on the wavelength, and inversely on the revisit time. According to this, L and C bands are well suited to detect and monitor this type of object, and therefore, to cover areas on the ground, which are generally lacking PS. Excluding the monitoring of urban areas and infrastructures, all other interferometric applications benefit from the possibility of using distributed reflectors. For instance, landslide monitoring could take great advantage from this opportunity, due to the relatively small size of the events, mostly occurring on hillsides or mountains with typically very few structures behaving as PS.

The characteristics of the S1 mission, or those of the future Tandem-L constellation, are very promising for exploiting distributed scatterers. In [10], the interferometric de-correlation times have been estimated for soils covered by low vegetation (pasture). Missions with revisit times shorter than the expected de-correlation times are suitable for use in such complex settings. The ALOS-2 mission improves the performance of the ALOS-PALSAR mission, but the nominal revisit time frequency is currently satisfied only on a few geographical areas, so that it is still difficult to find suitably populated datasets. The future Tandem-L constellation, with 2 satellites, 10 × 10 m^2^ resolution and an 8-day revisit time, promises greater potential, guaranteeing excellent values of interferometric coherence even on vegetated areas, and precision in displacement monitoring limited in the short term, but elevated over long time spans [30].

Many of the above considerations are evident from the analysis of the test case of Lesina Marina. As mentioned, the site is affected by the presence of sinkholes, as well as a very slow and spatially smooth uplift field, probably caused by the interaction between the water coming from an artificial canal and the underground soil, where gypsum with residual anhydride is present. The data at C-band and medium resolution from ERS and ENV are able to catch the large-scale uplift pattern, since the available observation time span is suitable to provide the required velocity precision. RSAT2 data improve the spatial density of detected targets, while, as foreseen by the model, S1 improves the C-band performance, by providing reliable estimation of the displacement rates in a limited time span. As expected, high-resolution data from CSK lead to a considerable increase of the PS spatial density, which improves the delineation of the spatial deformation pattern as clearly shown in Figure 4. Finally, high resolution and short revisit time data are also very promising for detecting small precursory terrain movements related to the sinkholes, although no such evidence could be found in the analyzed data.

**Figure 7:**
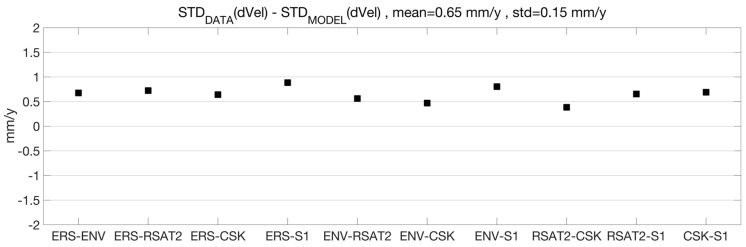
Difference between the standard deviation of the PS velocities difference from real data (Table 4), and the same standard deviation predicted by the model.

We also attempted a comparison between the outcomes of the model developed for assessing the MTI product precision, and the results obtained by processing the real datasets from ERS, ENV, RSAT2, CSK and S1 missions available for this test case. As sketched in plot C of Figure 1, the model predicts velocity dispersions below 1 mm/year for all datasets; therefore, we basically expect that products from different datasets would show similar levels of precision. Indeed, datasets with few images have long observation timespans that compensate for the lack of estimation points in the temporal fit. Moreover, independent in situ measurements were not available to us; these are essential for deriving the accuracy of the MTI velocity values to be compared to the model’s predictions. According to this, the only valuable approach to perform a comparison consists of using the velocity difference between datasets. Table 4 reports the standard deviation values of the velocity difference ($\sigma_{\Delta v}^{D}$) computed for all possible dataset couples by using the MTI products. The same quantities were estimated as $\sigma_{\Delta v}^{M}=\sqrt{\sigma_{v_{i}}^{2}+\sigma_{v_{j}}^{2}}$ by using the velocity standard deviations $\sigma_{v}$ computed through the model. Figure 7 shows the difference between $\sigma_{\Delta v}^{D}$ and $\sigma_{\Delta v}^{M}$: the values are below 1 mm/year for all the dataset combinations, with a mean of 0.6 mm/year. The model slightly overestimates the precision with respect to the values obtained from the MTI processing in a real and non-ideal scenario, where residual processing errors (possibly due to uncompensated atmospheric and/or orbital artifacts, for example) can contaminate the final estimation. Also, slight deviations from the assumed temporal constancy and linearity of the spatial uplift pattern could contribute to the mentioned differences.

## 5. Conclusions

The paper discussed the opportunities of MTI applications for ground instability monitoring, by assessing the performance of different available satellite missions, according to acquisition parameters. This performance analysis allows us to foresee, in the hypotheses of persistent scatterers, the quality of displacement maps estimated through MTI according to the mission characteristics, and thus may provide support for the SAR data selection, or for the definition of the geometrical and radiometrical configurations of future missions.

For instance, high-resolution data are found to increase the density of coherent targets, thus improving the monitoring of local scale events. Short X-band wavelengths improve the sensitivity to displacements. Short revisit times allow collecting large data stacks in short times, and improve the temporal sampling, thus increasing the chances of catching pre-failure signals (high-rate, nonlinear signals). The precision of displacement rate detection depends on the number of images and on the phase noise, while the precision of the residual height depends also on the orbital tube size. These relations are here formalized in a conceptual model, by assuming typical values for target characteristics such as persistent scatterer shape and signal to clutter ratio. The model allows us to derive quantitative, although indicative predictions for the performance of several missions (present and future), based on the above mentioned mission parameters.

In particular, for example, Sentinel-1 (S1), which will provide SAR data over the next years with short revisit times, is able to provide reliable displacement estimations at large scale, and in quite limited observation time spans (accuracy of about 1 mm/year for time series covering 1 year). However, the narrow orbital tube size, may limit the height precision, and consequently the geo-location quality (e.g., vertical precision above 20 cm with observation time span below 12 months, assuming S1-A/B 12 days repetitivity). X-band missions as COSMO-SkyMed (CSK) or TerraSAR-X, show the best performance in terms of precision on the estimated mean velocity and height, especially for limited observation time-spans (e.g., precision of 2 mm/year for velocity and 3 cm for height, with 12 months of observation time and 1 acquisition per month). By considering the forthcoming SAR missions (e.g., SAOCOM, Tandem-L, NISAR, RADARSAT Constellation Mission, PAZ, COSMO-SkyMED-SG, TerraSAR-X-NG), the resolutions and revisit times will be able to further improve the interferometric performances.

We also illustrated our findings by discussing an example of multi-sensor ground instability investigation performed over Lesina Marina, in Southern Italy, obtained by processing SAR datasets acquired from ERS, ENVISAT (ENV), RADARSAT2 (RSAT2), CSK and S1, and covering more than 20 years with varying ground resolutions, frequency bands and repeat times. Both sinkholes and a slow uplift field affect the site. Mean velocity maps and integrated displacement time series show that the uplift phenomenon has been going on rather steadily for the last 20 years. The different datasets provide values of spatial density of the detected targets, as well as the mean velocity precisions that depend on the specific mission, as predicted by the model. For instance, both RSAT2 and S1 data improve the spatial density of the measurements with respect to the ERS and ENV missions. S1 improves on the performances of C-band sensors by providing precise displacement estimations in a limited time span. The high-resolution data from CSK increases the PS spatial density considerably. Further investigations are ongoing, searching for evidence of small abrupt signals of displacements in order to demonstrate the potential of CSK and S1 for detecting small precursory terrain movements, such as those related to the sinkholes.

## Figures and Tables

**Table 1:** List of some satellite SAR missions and relevant parameters (updated from [3]).

Satellite Mission	Wave-Length (cm)	Life Status	Resolution Az./Range (m)	Repeat Cycle (days)	Swath Width (km)	Max. Vel. (cm/year)	Incident Angle (degree)
**L Band**							
**J-ERS**	23.5	1992÷1998	18	44	75	48.7	35
**ALOS PALSAR**	23.6	2006÷2011	≈ 5/(7÷88)	46	40÷70	46.8	8÷60
**ALOS-2**	22.9	2014	1/3 (3÷10)/(3÷10) 100/100	14	25 (Spot) 50÷70 (Strip) 350 (Scan)	149.2	8÷70
**SAOCOM** * (2 Sat)	23.5	2018	(10÷50)/(10÷50)	8, 16	20÷150	268 134	20÷50
**Tandem-L** * (2 Sat)	23.6	2023	≈(7÷10)/(7÷10)	8, 16	350	268 134	30÷50
**NISAR** * (L & S)	24 & 12	2021	6.4/(2÷6) (L) 6.9/(2÷30) (S)	12	>240 Km	180 (L) 90 (S)	33÷47
**C Band**							
**ERS-1/2**	5.6	1992÷2001	≈6/24	35	100	14.6	23
**ENVISAT**	5.6	2003÷2010	≈6/24	35	100	14.6	19÷44
**RADARSAT-1**	5.5	1995÷2013	≈(8÷30)/(8÷30)	24	45 (fine) 100 (Strip) 200 (Scan)	20.4	20÷50
**RADARSAT-2**	5.5	2007	≈3/3 ≈8/8 ≈26/25	24	10 (Spot) 40 (Strip) 200 (Scan)	20.4	20÷50
**Sentinel-1**	5.6	2014	20/5	6, 12	250	85 42.5	30÷46
**Gaofen-3**	5.6	2016	≈1/1 ≈25/25	29	10 (Spot) 130 (Strip)	17.6	20÷50
**RADARSAT Constellation Mission** * (3 Sat)	5.5	2018	(3÷50)/(3÷50)	4÷12	30÷350	42÷125	20÷55
**X Band**							
**COSMO-SkyMED** (4 Sat)	3.1	2007	1.0/1.0 ≈2.5/2.5 ≈30/30	2, 4, 8, 16	10 (Spot) 40 (Strip) 200 (Scan)	17.7 35.4 70.7 141.4	20÷60
**TerraSAR-X**	3.1	2007	1.0/1.0 ≈3.3/2.8 ≈20/20	11	10 (Spot) 30 (Strip) 100 (Scan)	25.7	20÷55
**KOMPSAT-5**	3.2	2013	3/3 1/1	28	5 (Spot) 30 (Strip)	10.4	20÷45
**COSMO-SkyMED-SG** * (2 Sat)	3.1	2020	(1÷3)/(1÷3)	8, 16	10÷40	17.7 35.4	20÷60
**TerraSAR-X-NG** * (constel. with PAZ)	3.1	2020	(0.25÷30)/(0.25÷30)	11	5÷20 (Spot) 10÷24 (Strip) 50÷400 (TOPS)	25.7	20÷50
**PAZ** (constel. with TerraSAR-X)	3.1	2018	(1÷6)/(1÷18)	11	10 (Spot) 30 (Strip) 100 (Scan)	25.7	20÷50

* Future missions.

**Table 2:** For each satellite mission in Figure 2, the table reports: acquisition mode, resolution in ground range (GR) and azimuth (Az), number of images (*N*), observation time span (*T)*, and spatial density of the coherent scatterers.

Mission	Res. GR/Az (m)	Inc. Angle (degree)	*N*	*T* [mm.yyyy, mm.yyyy]	Density (PS/km^2^)
ERS	20/5	23	24	[06.1995, 06.2000]	90
ENV	20/5	23	34	[10.2003, 12.2009]	75
RSAT2	12/5	33	40	[01.2009, 05.2012]	228
S1	5/20	34	49	[10.2014, 06.2016]	109
CSK	3/3	34	45	[05.2011, 08.2014]	1018

**Table 3:** Diameter values of the satellite orbital tube.

Mission	Orbital Tube Diameter (m)
ALOS2	1000
ENV	1000
RSAT2	2000
S1	100
CSK	2000

**Table 4:** Mean (m), standard deviation (std) and correlation coefficient (R) of the difference between velocities from the different datasets in Figure 3.

Mission	ENV	RSAT2	CSK	S1
ERS	m = −0.44 std = 0.87 R = 0.75	m = −0.19 std = 1.05 R= 0.77	m = −0.10 std = 0.89 R = 0.78	m = −0.21 std = 1.09 R = 0.67
ENV		m = 0.51 std = 0.86 R = 0.82	m = 0.37 std = 0.68 R = 0.88	m = 0.25 std = 0.96 R = 0.75
RSAT2			m = 0.14 std = 0.72 R = 0.89	m = 0.11 std = 0.96 R = 0.81
CSK				m = 0.13 std = 0.91 R = 0.82
